# Trace Elements in Leaf Extracts of Eucalyptus grandis Traditionally Used to Treat Common Cold and Flu

**DOI:** 10.5696/2156-9614-9.24.191214

**Published:** 2019-12-06

**Authors:** Artwell Kanda, France Ncube, Takudzwa K. Goronga

**Affiliations:** Department of Environmental Science, Bindura University of Science Education, Bindura, Zimbabwe

**Keywords:** eucalyptus, health risk, medicinal plants, mine tailings, phytoremediation, trace element, traditional medicinal remedy

## Abstract

**Background.:**

Eucalyptus species have been used for the remediation of mine tailings dams in Zimbabwe. However, a traditional medicinal remedy (TMR) for the treatment of mild acute respiratory infections, such as common cold and flu includes the use of Eucalyptus leaves.

**Objectives.:**

The aim of the present study was to determine total concentrations of selected potentially toxic trace elements (PTEs) in gold mine tailings and leaves of Eucalyptus grandis and to identify extractable fractions of PTEs in leaves via boiling for 10 minutes in water, which is the process used to create TMRs to treat common cold and flu.

**Methods.:**

Mine tailings and leaves of E. grandis were randomly collected at a gold mine tailings dam between April and June 2019. They were digested for laboratory analysis using standard analytical methods. Leaves were boiled in water for 10 minutes to prepare the TMR as practiced by the local community. The concentrations of PTEs were determined spectrometrically. Significant differences between PTEs in young and mature leaves were determined by analysis of variance.

**Results.:**

Mine tailings were acidic (pH 4.52±0.62) with very low content of organic matter (0.02%) and contained PTEs in increasing concentrations of cadmium (Cd) < nickel (Ni) < lead (Pb) < chromium (Cr) < copper (Cu) < zinc (Zn) (*n = 27*). Mature leaves of E. grandis had higher concentrations than young leaves for Cr, Pb and Zn (p <0.05) which were lower than permissible limits in medicinal plants. Overall, boiling leaves in water for 10 minutes resulted in low extraction of PTEs (< 20%).

**Participant Consent.:**

Obtained

**Conclusions.:**

Concentrations of PTEs in leaves and leaf extracts of E. grandis were very low. However, TMRs should not be prepared from medicinal plants growing on metalliferous environments, such as mine tailings dams, due to the presence of cumulative toxins such as Cd and Pb. Further studies are needed to investigate the effect of various boiling times and should include arsenic in the studied PTEs.

**Competing Interests.:**

The authors declare no competing interests for this study.

## Introduction

The use of medicinal plants is an ancient medical practice dating back to nomadic life ways in hunter-gatherer communities.^1^ Despite developments in science and biotechnology, plants remain a key source of traditional medicinal remedies (TMRs) for various ailments across socio-cultural settings.^2^ However, plants can accumulate potentially toxic trace elements (PTEs) from the environment.^3^ Many plants, including eucalyptus, are used to prepare TMRs for the treatment of common cold and flu.^4^,^5^ The leaves of eucalyptus have antibacterial, antifungal, analgesic and anti-inflammatory effects, along with antioxidative properties.^6^

Eucalyptus trees have a low to moderate rate of PTE accumulation in above-ground parts.^7^ The species gained attention for *in situ* remediation of mine sites in Zimbabwe. Revegetated mine tailings are exploited for non-timber products such as honey, fruits and herbs. Earlier studies showed that eucalyptus trees can accumulate PTEs from contaminated sites into their leaves.^8^,^9^Therefore, the consumption of TMRs made from leaves of eucalyptus growing in trace element-contaminated soil could be an important route of human exposure to PTEs. Potentially toxic trace elements (e. g. cadmium (Cd), chromium (Cr), copper (Cu), lead (Pb)) are harmful to human health.^10^,^11^

In the present study, the total concentrations of PTEs in gold mine tailings and leaves (young and mature) of Eucalyptus grandis growing on an inactive revegetated gold mine tailings dam (GMTD) were determined. The extractable fraction of PTEs after boiling leaves in water for 10 minutes (the process by which TMRs for the treatment of common cold and flu are made) was also determined.

## Methods

The study was performed at an inactive GMTD in northern Zimbabwe from April to June 2019. The tailings dam was revegetated with eucalyptus trees. Nine quadrants were randomly established on the dam's surface. Three sub-samples were collected at the surface from each quadrant and separately put into sealable polythene bags. Laboratory samples were prepared as described by Darus *et al*.^12^ Tailings were sieved (<2 mm), oven-dried (105°C, 24 hours), ground into powder and digested (0.500 g) in an acid mixture (37% hydrochloric acid: 65% nitric acid: water; 1: 1: 1 vol/vol; 3 ml) at 95°C until white fumes appeared. The digests (n = 27) were cooled, filtered (Whatman No. 42) and diluted to volume (50 ml) with deionized water. Separate oven-dried (<2 mm) samples (n = 27) were used for the determination of organic matter (OM) content and pH (calcium chloride).^13^,^14^

To determine the mass of leaves used, volume of water, and boiling time to prepare the TMR, local households (n = 20) familiar with the practice were purposively selected and consulted about typical practices. The selected households were educated about the purpose of the study, and gave informed consent for the results to be published. It was determined that leaves (9.56±1.34 g) are typically boiled in water (3004.35±79.65 ml) for 10.43±2.42 minutes. Households either added the leaves to water and brought the water to a boil (73.9%) or added the leaves to boiling water (26.1%). Five (5) E. grandis trees, about 4 m high, (16%) were randomly selected from the GMTD to sample leaves. They were typical trees whose leaves are used by the local community to make the TMR for the treatment of common cold and flu. Young and mature leaves were separately harvested using a stainless-steel scalpel and gloved hands above 1 m height off the ground. Three composites (young, mature) were separately made from the leaves from a single tree. Visibly diseased and pest-affected leaves were not sampled. Collected samples were kept in paper envelopes and preserved in polythene bags. At the laboratory, they were washed with deionized water. From each composite (young, mature leaves), duplicate sub-samples were made for the determination of total PTEs and hot water extractable concentrations of PTEs. For the determination of total PTE concentrations (mg/kg), sub-samples (9.56 g) were oven dried (90°C; 12 hours). A dried sample (0.500 g) was digested in 65% nitric acid and 30% hydrogen peroxide (5:1; vol/vol) at 150°C over a hot plate in a fume hood.^15^ Deionized water (10 ml) was added to the cooled digests which were filtered (Whatman No. 42) and diluted to 20 ml with deionized water to give 30 leaf sample solutions.

**Abbreviations**
*GMTD*: Gold mine tailings dam
*OM*: Organic matter
*PTEs*: Potentially toxic trace elements
*TMRs*: Traditional medicinal remedies

To determine the concentrations of PTEs extracted from leaves in hot aqueous solution (mg/kg), fresh harvested young or mature leaves (9.56 g) were added to 300 ml deionized water and boiled on a hot plate (at 500°C) for 10 minutes. The solutions (n = 30) were separately concentrated by evaporation to about 60 ml, cooled, filtered and then diluted to 100 ml with deionized water. The concentration was expressed as a percentage of the total in the leaf. Tailings, leaf digests and hot water extracts were analyzed for Cd, Cr, Cu, nickel (Ni), Pb and zinc (Zn) using inductively coupled plasma - optical emission spectroscopy (Thermo Scientific iCAP 6500; England). The estimated quantity of PTE taken in by consuming 300 ml of the TMR was taken as the hot water-extractable concentration obtained by boiling (9.56 g in 300 ml water) expressed in μg/day assuming the volume was taken once per day.

Significant differences between the concentration of a PTE in mature and young leaves for a given tree were investigated using independent *t*-test. All analyses were performed using the Statistical Package for the Social Sciences (SPSS) version 23, at a 95% level of confidence and considered significantly different at p <0.05.

## Results

Table 1 shows the variation of measured parameters for gold mine tailings. Results indicate that the tailings were acidic (pH 4.52±0.62) with very low OM content (0.02%), and contained Cd, Cr, Cu, Ni, Pb and Zn. Overall, the total concentrations of PTEs in mine tailings appeared to increase in the order: Cd < Ni < Pb < Cr < Cu < Zn.

**Table 1:** Total Concentrations of Trace Elements, pH and Organic Matter in Mine Tailings

**Quadrant**	**Statistic**	**Cd**	**Cr**	**Cu**	**Ni**	**Pb**	**Zn**	**pH**	**OM (%)**
1	Mean	0.030	1.302	1.284	0.264	0.723	5.000	3.83	0.014
	SD	0.004	0.136	0.070	0.023	0.139	1.621	0.04	0.003
2	Mean	0.046	2.427	4.379	0.388	1.565	3.348	4.05	0.030
	SD	0.010	0.455	1.147	0.115	0.344	0.450	0.06	0.003
3	Mean	0.027	1.364	4.367	0.518	1.871	6.848	4.23	0.021
	SD	0.004	0.359	1.200	0.142	0.240	1.569	0.04	0.004
4	Mean	0.010	2.080	1.742	0.359	0.755	7.464	4.47	0.075
	SD	0.017	0.754	0.466	0.038	0.107	1.416	0.04	0.08
5	Mean	0.025	2.191	4.038	0.678	0.995	2.701	5.29	0.046
	SD	0.004	0.550	1.197	0.129	0.191	0.315	0.06	0.010
6	Mean	0.025	2.533	1.629	0.975	1.499	4.858	4.27	0.032
	SD	0.006	0.534	0.622	0.581	0.239	1.524	0.04	0.008
7	Mean	0.039	2.171	3.176	0.714	1.916	3.150	3.96	0.018
	SD	0.009	0.489	0.861	0.782	0.341	0.219	0.07	0.002
8	Mean	0.016	1.142	2.102	0.588	0.688	4.915	4.84	0.012
	SD	0.014	0.064	0.676	0.065	0.018	0.561	0.07	0.03
9	Mean	0.020	1.965	3.647	0.327	1.556	2.600	5.75	0.027
	SD	0.026	0.203	0.527	0.102	0.322	0.569	0.06	0.007

Concentrations are expressed as means of triplicate samples (mg/kd, dry weight)

Abbreviation: SD, standard deviation.

### Total and extractable concentrations of potentially toxic trace elements in leaves of E. grandis

The total concentrations of PTEs in young and mature leaves of E. grandis growing on a revegetated GMTD are presented in Table 2. Results suggest that E. grandis accumulated Cd, Cr, Cu, Ni, Pb and Zn into its leaves. Further, the overall (*n = 15*) total concentrations of Cr, Pb and Zn were higher in mature leaves than in young leaves (p <0.05). Total concentrations of PTEs appeared to increase in young leaves in the order: Cd < Pb < Cr < Ni < Cu < Zn and in mature leaves: Cd < Ni < Cr < Pb < Cu < Zn. Values were lower than the Food and Agriculture Organization of the United Nations/World Health Organization (FAO/WHO) permissible limits in medicinal plants described for different countries *(Table 2).*

**Table 2:** Total Concentrations of PTEs in Leaves of E. grandis (mg/kg, dry weight) and Extractable Fractions (%)

**Tree**	**Leaf**	**Statistic**	**Cd**	**Cr**	**Cu**	**Ni**	**Pb**	**Zn**
1	Young	Mean	0.0083 (0)	0.0744^*^ (0)	1.2844 (9.31)	0.1098^*^ (19.22)	0.0881^*^ (0)	2.0000^*^ (7.24)
		SD	0.0102	0.0277	0.1343	0.0206	0.0271	0.4233
	Mature	Mean	0.0188 (0)	0.1524^*^ (0)	0.9120 (6.37)	0.1252^*^ (20.60)	0.3057^*^ (5.36)	3.2281^*^ (9.08)
		SD	0.0209	0.0389	0.3317	0.0209	0.0479	0.3827
2	Young	Mean	0.0021^*^ (0)	0.0795 (0)	1.5465 (8.61)	0.1140^*^ (17.98)	0.0705^*^ (0)	1.6412^*^ (6.37)
		SD	0.0007	0.0197	1.1619	0.0448	0.0188110.5564	
	Mature	Mean	0.0121^*^ (0)	0.0950 (0)	0.6792 (0)	0.1598^*^ (22.28)	0.2000^*^ (3.30)	4.8027^*^ (10.68)
		SD	0.0031	0.0128	0.1958	0.0151	0.0204	0.4156
3	Young	Mean	0.0026^*^ (0)	0.0746 (0)	1.0464 (6.89)	0.0800^*^ (0)	0.1101^*^ (0)	1.6010^*^ (6.61)
		SD	0.0010	0.0132	0.1942	0.0142	0.0296	0.7031
	Mature	Mean	0.0122^*^ (0)	0.1947 (26.19)	0.9772 (6.02)	0.0955^*^ (0)	0.2609^*^ (4.48)	4.5244^*^ (10.74)
		SD	0.0031	0.0872	0.2847	0.0151	0.0189	1.5425
4	Young	Mean	0.0145 (0)	0.0641^*^ (0)	1.3075^*^ (7.51)	0.0645^*^ (0)	0.0534^*^ (0)	1.0471^*^ (0)
		SD	0.0229	0.0576	0.1389	0.0157	0.0464	0.2611
	Mature	Mean	0.0045 (0)	0.1706^*^ (0)	0.7533^*^ (4.08)	0.1074^*^ (20.11)	0.1792^*^ (2.85)	3.6145^*^ (8.88)
		SD	0.0011	0.0339	0.1769	0.0184	0.0362	0.6132
5	Young	Mean	0.0032 (0)	0.0945^*^ (0)	1.0498 (6.87)	0.1070^*^ (18.88)	<0.005^*^ (0)	0.8643^*^ (0)
		SD	0.0019	0.0217	0.2852	0.0299	-	0.1760
	Mature	Mean	0.0077 (0)	0.1960^*^ (26.22)	0.6608 (0)	0.1314^*^ (21.46)	0.0864^*^ (0)	2.7536 (7.95)
		SD	0.0054	0.0330	0.2205	0.0394	0.0163	0.4355
Overall (*n=15*)	Young	Mean	0.0061 (0)	0.0774^a^ (0)	1.2469^a^ (7.95)	0.1071 (11.58)	0.0633^a^ (0)	1.4308^a^ (4.97)
		SD	0.0167	0.0289	0.2517	0.0452	0.0456	0.5832
	Mature	Mean	0.0111 (0)	0.1617^b^ (12.68)	0.7965^b^ (3.70)	0.1239 (17.92)	0.2064^b^ (3.88)	3.7847^b^ (9.73)
		SD	0.0097	0.0559	0.2477	0.0303	0.0816	1.0517
Permissible concentration in medicinal plants16			0.3	2	20 - 150	1.63	10	50

^*^ Significantly different total concentrations of a PTE between young and mature leaves for a given tree (p <0.05).

Different superscripts; a, b denote significantly different overall total concentrations of a PTE between young and mature leaves (p <0.05).

Values in parentheses are fractions of the total PTE concentration in leaves (%) extracted by boiling them in water for 10 minutes.

Abbreviation: SD, standard deviation.

The proportions (%) of PTEs extracted by boiling leaves of E. grandis in water for 10 minutes are shown in Table 2. The results show that Cd, Cr and Pb were not extracted (0%) from young leaves. Nickel was the only fraction of PTE extracted from leaves (*n = 15*) that was not significantly different between young and mature leaves (p >0.05). Overall, extraction of PTEs in young leaves increased in the order: Cd = Cr = Pb (0%) < Zn < Cu < Ni, ranging from 0 to 11.582% and Cd < Cu < Pb < Zn < Cr < Ni for mature leaves (range 0 to 17.92%). For mature leaves, Cu, Pb and Zn were below 10%, while Cr and Ni were greater than 10 but less than 20%.

Table 3 shows the amount of PTEs in the prepared TMR made by boiling 9.56 g of E. grandis fresh leaves in 300 ml of water for 10 minutes. The results indicate that TMR made from young leaves did not contain Cr, Cd and Pb in detectable amounts, but had a higher concentration of Cu than TMR made from mature leaves. The TMR prepared from mature leaves contained more Cr, Pb and Zn than TMR prepared from young leaves (p <0.05).

**Table 3:** Estimated Daily Intake of Potentially Toxic Elements (μg/day) in the Traditional Medicinal Remedy (9.56 g in 300 ml water) for the Treatment of Cold and Flu

	**Cd**	**Cr**	**Cu**	**Ni**	**Pb**	**Zn**
Young leaves	ND	ND^*^	0.095^*^	0.119	ND^*^	0.055^*^
Mature leaves	ND	0.196^*^	0.003 ^*^	0.212	0.008^*^	0.352^*^

^*^ Significantly different concentrations (down a column) for a PTE between young and mature leaves (p <0.05).

Abbreviations: ND, not detected.

## Discussion

Our results are consistent with previous findings that gold mine tailings are usually acidic, have low OM and are laden with PTEs.^17^,^18^ Low pH and OM generally favor the release of PTEs by soil, such as tailings, and eventual uptake by plants.^19^ Differences in the mineralogy of mined ore, extraction and natural biogeochemical processes may explain the variability of the concentrations of PTEs in tailings.^20^ While Cr, Cu and Zn are essential elements, Cd and Pb are non-essential.^21^ Results show very low concentrations of PTEs in tailings compared to similar studies. Revegetation of the GMTD, mineral ore processing and tailings management could explain their low concentrations in the present study.

### Concentrations of potentially toxic trace elements in leaves of E. grandis growing on a gold mine tailings dam

To the best of the authors' knowledge and available literature, there have been no studies of the use of E. grandis growing on tailings dams for medicinal purposes. Nevertheless, the species is widely used for medicinal purposes. The findings of the present study are consistent with several previous reports that eucalyptus species accumulate some PTEs in leaves.^7^,^22^,^23^ Children who consume these TMRs may be at higher risk than adults, especially in the winter season when the common cold and flu occur more frequently. The physiology of the plant and PTE characteristics may explain the greater accumulation of Cr, Pb, and Zn in mature leaves compared to young leaves. In a study of heavy metal content of tea leaves in Puan County, China, by Zhang *et al*., Cr, Pb and Zn concentrations were higher in mature leaves compared to young leaves.^24^ However, Doganlar *et al*. reported higher concentrations for Cu, Pb and Zn in E. camaldulensis sampled from urban parks and streets of Adana city in Turkey.^22^ Variations in concentrations could be explained by the total PTE in the tailings, leaves and tailings characteristics such as pH and OM, which influence their bioavailability. The results suggest no potential safety risk in using the leaves of E. grandis, as concentrations were much lower than maximum allowable limits in medicinal plants.

### Extractable fraction of total potentially toxic trace elements from leaves of E. grandis

The concentrations of PTEs in hot water extracts prepared from medicinal plants are affected by the OM content that can chelate them, solubility of each PTE and the quality of the water used.^25^ Furthermore, the extraction of PTEs from leaves appeared to depend on their total concentrations and leaf maturity. The non-essential trace elements, Cd and Pb, were poorly extractable compared to Ni and Cr. This could be an important property in the use of medicinal formulations requiring hot water extraction. Assessments of the safety of medicinal plants should mimic how they are used in practice and avoid reporting total concentrations where water extracts are used, as such reports give overestimated PTE intake values.

The species E. grandis extracts PTEs from the tailings into its leaves in very small quantities, resulting in low hot water extraction coefficients. Generally, mature leaves are used for the preparation of the TMR for the treatment of the common cold and flu. The presence of Pb in the aqueous solution is cause for concern, especially for children who frequently get the common cold and flu and use TMRs. Findings from the present study suggest that the intake of TMR prepared from leaves of E. grandis presented no potential health risk due to Cd and Pb. Furthermore, the TMR is not consumed as part of a diet but a remedy: that is, not consumed daily, but only as needed. Trace elements (Cr, Cu and Zn) are essential for human metabolism. Their concentrations were much lower than dietary daily requirements.^26^ Although the concentrations of PTEs were very low in the present study, they may be cumulative over time. Our results are generalizable to many low-income areas, especially in Africa, where mine sites are revegetated with eucalyptus trees which are eventually exploited for non-timber products.

## Conclusions

The tailings at the studied GMTD contained PTEs which accumulated in leaves of E. grandis in trace amounts. Boiling the leaves in water resulted in low extraction of PTEs. Medicinal plants may contribute to the body's requirement of essential elements such as Cr, Cu and Zn. However, the use of TMRs made from E. grandis growing on metalliferous tailings dams may constitute a health risk due to the presence of cumulative toxic elements such as Pb. Formulations of TMRs should not be prepared from any parts of medicinal plants harvested from metalliferous habitats such as mine tailings dams.
